# Semipermeable Capsules Wrapping a Multifunctional and Self-regulated Co-culture Microenvironment for Osteogenic Differentiation

**DOI:** 10.1038/srep21883

**Published:** 2016-02-24

**Authors:** Clara R. Correia, Rogério P. Pirraco, Mariana T. Cerqueira, Alexandra P. Marques, Rui L. Reis, João F. Mano

**Affiliations:** 1 3B’s Research Group – Biomaterials, Biodegradables and Biomimetics, University of Minho, Headquarters of the European Institute of Excellence on Tissue Engineering and Regenerative Medicine, AvePark, 4805-017 Barco, Guimarães, Portugal; 2 ICVS/3B’s – PT Government Associate Laboratory, Braga/Guimarães, Portugal

## Abstract

A new concept of semipermeable reservoirs containing co-cultures of cells and supporting microparticles is presented, inspired by the multi-phenotypic cellular environment of bone. Based on the deconstruction of the “stem cell niche”, the developed capsules are designed to drive a self-regulated osteogenesis. PLLA microparticles functionalized with collagen I, and a co-culture of adipose stem (ASCs) and endothelial (ECs) cells are immobilized in spherical liquified capsules. The capsules are coated with multilayers of poly(L-lysine), alginate, and chitosan nano-assembled through layer-by-layer. Capsules encapsulating ASCs alone or in a co-culture with ECs are cultured in endothelial medium with or without osteogenic differentiation factors. Results show that osteogenesis is enhanced by the co-encapsulation, which occurs even in the absence of differentiation factors. These findings are supported by an increased ALP activity and matrix mineralization, osteopontin detection, and the up regulation of *BMP-2, RUNX2* and *BSP*. The liquified co-capsules also act as a VEGF and BMP-2 cytokines release system. The proposed liquified capsules might be a valuable injectable self-regulated system for bone regeneration employing highly translational cell sources.

The successful regeneration of bone remains a significant challenge in orthopedic research. Strong affords have been made as a potential alternative to the conventional use of bone grafts due to their limitless supply and no disease transmission. In particular, vascularization remains an obstacle to engineer large volume bone tissues. In order to face such challenges, new generations of devices for tissue engineering (TE) should improve the physical and biochemical cues operating in tandem during native regeneration, in particular at the scale/organizational-level of the stem cell niche. The understanding and the deconstruction of these factors (e.g. multiple cell types exchanging both paracrine and direct signals, structural and chemical arrangement of the extracellular matrix, mechanical signals) should be then incorporated into the design of advanced and clinically relevant biomimetic biomaterials^1^. Moreover, biomimetic biomaterials have been increasingly combined with multi-phenotypic cells to produce new advanced systems that better model the complex cellular environment of natural tissues. Particularly, considering the highly vascularized network of bone, osteoprogenitor cells inevitably interact with endothelial cells (ECs) in a synergetic fashion^2^,^3^. The cellular interactions facilitate viability and proliferation as well as generation of growth factors and proteins that promote osteogenic and angiogenic differentiation, while enabling other key biological tasks during bone development^4^,^5^. Different fundamental studies have established that a reciprocal interaction, both physical and biochemical, between osteoblasts, stem cells and endothelial cells occurs during osteogenesis, as well as the 2D interaction between endothelial and stem cells towards osteogenic differentiation was already demonstrated *in vitro* and *in vivo*^6^,^7^,^8^,^9^,^10^,^11^,^12^,^13^. While endothelial cells secrete numerous regulatory molecules that influence the differentiation and activity of bone forming cells^14^,^15^,^16^,^17^, osteoblasts/osteoprogenitor cells release diverse pro-angiogenic growth factors^7^,^18^,^19^,^20^. However, the commonly used co-culture systems involve the challenge of generating stable *in vitro* pre-vasculature in the constructs with typically a slow rate of anastomosis efficacy. Moreover, conventional porous hybrid scaffolds have typically a fixed geometry and need to be implanted under open surgery. Alternatives to transport biomaterials and cells are injectable systems that could carry all the necessary cargo able to stimulate upon implantation the formation of new bone vascularized tissue using minimally invasive procedures^21^. To achieve such concept, one could envisage the development of an injectable device, pre-cultured with cells that could have all the necessary instructive signals to upon implantation generate new bone tissues and an initial support for angiogenesis that is expected to integrate with time with the surrounding vasculature. Considering the referred requirements, we propose a rather unique combination of functional biomaterials and cells for the groundbreaking advances of engineering self-regulated 3D compartmentalized devices. We aim to transfigure the concept of conventional 3D scaffolds for TE, typically associated on the use of porous structures or hydrogels to support cells, by using an alternative hierarchical methodology in which solid microparticles and cells are wrapped by semipermeable capsules. Our previous studies demonstrated already the suitability of the liquified capsules as successful bioencapsulation devices^22^,^23^. In this work, inspired by the native co-existence of multiple cell types and from the concept of deconstructing the “stem cell niche”^1^, we propose for the first time to use liquified capsules as self-regulated co-encapsulation reservoirs of stem and endothelial cells. Owing to a number of appealing features, such as being available in large quantities with diminutive donor site morbidity or patient discomfort, adipose tissue was the source used to isolate both cell types. Figure 1 represents the methodology of the production of the capsules. Capsules are composed by three essential components: *(i)* a permselective membrane barrier that wraps the liquefied core of the capsule, ensuring permeability to essential molecules for cell survival, flexibility to the capsule, and enhancing direct contact between the encapsulated materials; *(ii)* surface functionalized collagen I poly(L-lactic acid) (PLLA) microparticles as cell adhesion sites; and *(iii)* a co-culture of adipose stem (ASCs) and endothelial (ECs) cells. The membrane of the capsules is produced using the layer-by-layer assembly. Although many interactions may be employed for the build-up of the multilayers^24^, we employed simple electrostatic forces for assembling oppositely charged polyelectrolytes. Our main hypothesis is that ECs would provide a more physiologically relevant microenvironment as well as regulate the structure and function of ASCs towards the osteogenic lineage (ASCs have been shown in numerous studies to exhibit the potential to contribute to chondrogenesis, osteogenesis, adipogenesis, myogenesis as well as some aspects of neurogenesis^25^,^26^,^27^,^28^). Therefore, we expect that ECs would led ASCs to differentiate without requiring the supplementation of two main osteogenic differentiation factors, namely dexamethasone and ascorbic acid. Moreover, we expect that the liquefied environment of the capsules will assure the excellent diffusion of nutrients to the encapsulated cells, even those at the inner region of the capsule, and spatial freedom for cell communication and self-organization. The biodegradable nature of the capsules, combined with their intrinsic osteo- and angiogenic natures, could engender a novel generation of injectable biomimetic systems with clinically viability to be used in orthopedic applications. To test our hypothesis, the multifunctional liquified capsules encapsulating only ASCs (MONO capsules) or a co-culture with ECs (CO capsules) were cultured in endothelial medium with (EDAG medium) or without (EG medium) osteogenic differentiation factors.

## Materials and Methods

### Cells Isolation from Adipose Tissue

Subcutaneous adipose tissue from liposuction procedures was used to isolate both human adipose stem cells (ASCs)^29^ and human adipose microvascular endothelial cells (ECs)^30^. The collected tissues were obtained under a cooperation agreement between the 3B’s Research Group and Hospital da Prelada (Porto, Portugal), after approval of the Competent Ethics Committee (CEC). The human tissues received were handled in accordance with the guidelines approved by the CEC. Informed consent was obtained from all subjects. Briefly, samples were transported in phosphate-buffered saline (PBS, Sigma-Aldrich) supplemented with penicillin-streptomycin (10%, pen-strep, Gibco) and kept at 4 °C. The lipoaspirates were washed with PBS and incubated with collagenase type II A (0.05% w/v, Sigma-Aldrich) for 45 minutes at 37 °C in a shaking water bath. The digested samples were filtered (200 μm) and centrifuged at 800 g for 10 min at 4 °C. The obtained stromal vascular fraction (SVF) was resuspended in erythrocyte lysis buffer at pH 7.4 containing ammonium chloride (155 mM, Merk), potassium bicarbonate (5.7 mM, Riedel-de-Häen), and ethylenediaminetetraacetic acid (0.1 M, EDTA, Sigma-Aldrich) in distilled water. After 10 min of incubation at room temperature (RT), the mixture was centrifuged at 300 g for 5 minutes. To isolate the ASCs the red blood cell-free SFV was resuspended in α-MEM medium (Invitrogen) supplemented with fetal bovine serum (10%, FBS, Invitrogen) and pen-strep (1% v/v). To isolate the ECs the red blood cell-free SFV was resuspended in EndoGro^TM^-MV-VEGF complete media kit (Millipore Iberica). The ECs were plated in cell culture flasks, previously coated with gelatin (0.7% w/v, porcine skin type A, Sigma-Aldrich) for 30 min at 37 °C. Culture medium was changed 48 h after initial plating and then every 3-4 days for both cell phenotypes.

### Microparticles Production and Surface Functionalization

PLLA microparticles were produced by oil/water (o/w) emulsion technique and surface modified by combining plasma treatment with collagen I as similarly described in our previous reports^22^,^23^. PLLA (5% w/v, Mw~1,600–2,400, 70% crystallinity, Polysciences) was dissolved in methylene chloride (CH_2_Cl_2_, Fisher Chemical). Under agitation, this solution was added to polyvinyl alcohol (0.5%, PVA, Sigma-Aldrich). After 2 days at RT, the produced microparticles were subsequently collected, washed with distilled water, and lyophilized (Cryodos, Telstar). Microparticles were then placed in a plasma reactor chamber (PlasmaPrep5, Gala Instrumente) fitted with a radio frequency generator. Air was used as the working atmosphere. A glow discharge plasma (0.2mbar, 30V) was created for 15 min. Subsequently, microparticles (500 mg) were immersed in collagen I (1200 μg, rat protein tail, Gibco) diluted in acetic acid (0.02 M) for 4 h at RT.

### Mono- and Co-cultures Set Up

At 90% confluence, ASCs (passage 2) and ECs (passage 4–6) grown in T150 tissue culture flasks were washed with PBS and subsequently detached using TrypLE^TM^ Express solution (Life Technologies) at 37 °C for 5 min. PBS was added and the cell suspensions were centrifuged for 5 min at 300 g. Low viscosity sodium alginate from brown algae (1.5% w/v, ALG, ~250cP, Sigma-Aldrich) was dissolved in sodium chloride (0.15 M, NaCl, Enzymatic) containing MES hydrate (25 mM, Sigma-Aldrich) at pH 7. The solution was sterilized by 0.22 μm filtration. 5 × 10^6^ ASCs (5 × 10^6^.mL^−1^ of alginate) alone or mixed with ECs (5 × 10^6^.mL^−1^ of alginate, co-culture ratio 1:1) were added to the microparticles (50 mg.mL^−1^ of alginate). The alginate solutions were then used to produce liquified capsules encapsulating ASCs alone (MONO capsules) or in co-culture with ECs (CO capsules).

### Liquified Multilayered Capsules Production

All the conditions used to produce the liquified capsules (the combination of the polyelectrolytes, concentration, pH, number of layers, and time of adsorption and liquefaction) were optimized in our previous studies^22^,^23^. The alginate solutions containing cells and microparticles were added drop wise using a 21G needle to calcium chloride (0.1M, CaCl_2_, VWR) buffered with MES hydrate (25 mM, Sigma-Aldrich) at pH 7. After 20 min at RT, alginate hydrogels were collected and rinsed in a washing solution of NaCl (0.15M). The external membrane was processed by subsequent adsorption of oppositely charged polyelectrolytes by layer-by-layer deposition. Alginate hydrogel particles were first immersed in a poly-L-lysine (PLL, Mw~30,000–70,000, pH 7, Sigma-Aldrich), and subsequently in ALG solution (pH 7), water-soluble highly purified chitosan (CHT, pH 6, Protasan UP CL 213, viscosity 107 mPa.s, Mw = 2.7 × 10^5^ g.mol^−1^, 83% degree of deacetylation, NovaMatrix), and, ultimately, in ALG solution again. The polyelectrolyte solutions (0.5 mg.mL^−1^) were dissolved in NaCl (0.15M) containing MES hydrate (25 mM). Following a 10 min period for each polymer adsorption, the excess of macromolecules was removed by immersion in NaCl (0.15M) for 5 min. This process was repeated three times in order to obtain a 12-layered membrane. The coated hydrogels were immersed in EDTA (20 mM) at pH 7 for 5 min to liquefy the alginate core. The two formulations of liquified capsules obtained were cultured in EndoGro^TM^-MV-VEGF complete kit media without ascorbic acid (E). To the E medium, β-glycerophosphate (10 mM, G, Sigma-Aldrich) were added, here termed as EG medium. Additionally, capsules were also cultured in E medium containing all the osteogenic differentiation factors, namely, dexamethasone (10 nM, D, Sigma-Aldrich), ascorbic acid (50 μg.mL^−1^, A), and β-glycerophosphate (10 mM, G, Sigma-Aldrich), here termed as EDAG medium. Capsules were transferred to non-adhesive 24-well plates and incubated at 37 °C in a humidified 5% CO_2_ air atmosphere. The entire procedure was performed under sterile conditions. All the solutions used at this step were sterilized by filtration through a 0.22 μm filter.

### Flow Cytometry Analysis

The phenotypic profile of ASCs and ECs was assessed regarding mesenchymal (CD105-FITC, Arium; CD90-APC, and CD73-PE, BD Biosciences) and endothelial (CD31-APC, Citomed) markers before and after encapsulation within MONO and CO capsules. Isolated and encapsulated cells were harvested by TrypLE^TM^ Express solution (Life Technologies) at 37 °C for 5 min, and centrifuged. Samples were resuspended in PBS solution containing bovine serum albumin (3% w/v, BSA, Sigma-Aldrich) and the specified antibodies at the dilutions defined by the manufacture’s specifications. After 20 min at RT, samples were subsequently washed with PBS, centrifuged, fixed in PBS with formaldehyde (1% v/v, Sigma-Aldrich), and analyzed in a flow cytometer (BD FACSCalibur, CellQuest v3.3 software, BD Biosciences).

### Lipophilic Fluorescent Labeling

Prior to encapsulation, ASCs and ECs were incubated with the lipophilic dyes 3,3′-dioctadecyloxacarbocyanine perchlorate (DIO, green) and 1,1′-dioctadecyl-3,3,3′,3′-tetramethylindocarbocyanine perchlorate (DIL, orange), respectively. Cells were incubated with each dye (1 mL, 2 μM per 1 × 10^6^ cells) at 37 °C for 10 min. Labeled cells in the MONO and CO capsules were visualized by confocal microscopy (TCS SP8, Leica).

### Mitochondrial Metabolic Activity Quantification

Mitochondrial metabolic activity quantification was performed using a MTS colorimetric assay (CellTiter96^®^ AQueous one solution cell proliferation assay, Promega) according to manufacture’s specifications. Capsules (n = 4 per well in triplicate) were incubated with the reagent kit (1 mL per well) at 37 °C protected from light. After 3 h, absorbance was read at 490 nm using a microplate reader (Gen 5 2.01, Synergy HT, Biotek).

### Cell Proliferation Quantification

Total DNA quantification was performed after cell lysis (Quant-iT^TM^ PicoGreen^®^ dsDNA assay kit, Life Technologies). Samples (n = 4 per well in triplicate) were suspended in ultra-pure sterile water (1 mL per well). After 1 h in a 37 °C shaking water bath, samples were frozen at −80 °C overnight. Samples were defrosted and used according to the specifications of the kit. A standard curve for DNA analysis was generated with the provided dsDNA solution. After 10 min of incubation at RT, fluorescence was read at an excitation wavelength of 485/20 nm and 528/20 nm of emission using a microplate reader (Gen 5 2.01, Synergy HT, Biotek).

### Imaging Cell Morphology by Scanning Electron Microscopy (SEM)

Samples (n = 4 per well in triplicate) were washed with PBS and fixed at RT in formalin (10% v/v). After 1 h, samples were dehydrated in an increasing gradient series of ethanol for 10 min each. The membrane of capsules was destroyed to expose the core contents. After gold sputtering, samples were visualized by SEM (15 kV, JSM-6010LV, Jeol).

### Alkaline Phosphatase Activity Quantification

The activity of alkaline phosphatase was determined by the amount of *p*-nitrophenol. A substrate solution (pH 9.8) was prepared by dissolving 4-nitrophenylphosphate disodium salt hexahydrate (0.2% w/v, Sigma-Aldrich) in diethanolamine (1M, Sigma-Aldrich). Each sample (20 μL, in triplicate) was mixed with the prepared substrate solution (60 μL). After 45 min at 37 °C protected from light, the reaction was stopped (80 μL) with NaOH (2 M) and EDTA (0.2 mM). A standard curve with a range of concentrations was prepared by diluting 4-nitrophenol solution (10 mM, Sigma-Aldrich) in the stop solution. Absorbance was read at 405 nm in a microplate reader (Gen 5 2.01, Synergy HT, Biotek). Results were normalized with dsDNA quantification data.

### Calcium Quantification

Samples were washed with PBS and resuspended in 100 μL of HCl (6 M). A standard curve was prepared with a CaCl_2_ solution (5 mM). 10 μL of each sample and standard (in triplicate) were transferred to a 96-well plate. Upon mixing the calcium kit components according to manufacture’s specifications (Roche), absorbance was read at 570 nm in a microplate reader (Gen 5 2.01, Synergy HT, Biotek).

### Histological Mineralization Analysis

MONO and CO capsules cultured in EG or EDAG media were collected after 21 days, washed in PBS, and processed in an automatic spin tissue processor (STP120-2, Microm) for histological analysis. Afterwards, samples were embedded in paraffin and cut into sections of 5 μm thickness using a microtome (HM355S, Microm). To visualize calcium-rich deposits, sections were deparaffinized and subsequently immersed in alizarin red staining solution for 5 min at RT. Samples were then mounted and analyzed (Axio Imager Z1m, Zeiss).

### Immunofluorescence Staining on 3D structures and histological sections

Samples were fixed in formalin (10% v/v, BD Biosciences) for 30 min at RT. After fixation, capsules were destroyed and cells were permeabilized for 5 min at RT with Triton-X (0.1% v/v, Sigma-Aldrich). Samples were immersed in BSA (3% w/v) for 30 min at RT to block non-specific binding and then incubated overnight at 4 °C with the primary antibody rabbit anti-human osteopontin (Promega, 1:100 in 1% BSA). After PBS washing, samples were incubated for 1 h at RT with the secondary antibody anti-rabbit AlexaFluor 488 (BD Biosciences, 1:500 in 1% BSA). The immunofluorescence staining in the histological sections was performed in CO capsules cultured in EG or EDAG media for 21 days. Samples were washed in PBS and processed in an automatic spin tissue processor (STP120-2, Microm) for histological analysis. Afterwards, samples were embedded in paraffin and cut into sections of 5 μm thickness using a microtome (HM355S, Microm). After the deparaffinization process, the antigen retrieval was performed in a microwave-heated sodium citrate buffer (10 mM, Sigma-Aldrich). Samples were immersed in hydrogen peroxide (3% v/v, VWR) at RT for 30 min. The same immunofluorescence staining protocol used for the 3D structures was repeated with the adding of the primary antibody mouse anti-human CD31 (Sigma-Aldrich, 1:100 in 1% BSA) conjugated with the osteopontin and the secondary antibody anti-mouse AlexaFluor 594 (BD Biosciences, 1:500 in 1% BSA). Ultimately, 3D and 2D structures were counterstained with DAPI (Sigma-Aldrich, 1 mg.mL^−1^ diluted 1:1000 in PBS) for 5 min at RT and analyzed by confocal microscopy (TCS SP8, Leica).

### Quantification of Cytokines

The supernatants (500 μL) were stored at −80 °C with a diluted protease inhibitor cocktail (1:100, Sigma-Aldrich) until analysis. Commercially available human BMP-2 and VEGF ELISA development kits (Peprotech) were used according to manufacture’s specifications. Absorbance was read at 405–650 nm in a microplate reader (Gen 5 2.01, Synergy HT, Biotek).

### RNA Extraction and cDNA Production

The extraction of mRNA was performed using TRIzol reagent (Sigma-Aldrich). Capsules (n = 5 per well in triplicate) were stored in TRIzol (800 μL) at −80 °C until analysis. Samples were slowly defrosted on ice and chloroform (160 μL, Sigma-Aldrich) was added. After 15 min at 4 °C, samples were centrifuged at 13,000 rpm (Bioline, MCF-2360) for 15 min. The aqueous part of each sample was collected and isopropanol (400 μL, Sigma-Aldrich) was added. Following an overnight incubation at −20 °C, samples were centrifuged at 13,000 rpm for 10 min at 4 °C. The supernatant was discarded and the resulting pellets were washed with ethanol (70% v/v) in RNase/DNase free distilled water (VWR). The pellets were let to partially dry on air, and dissolved in RNase/DNase free distilled water (10 μL). RNA quantity and purity was determined in nanodrop spectrophotometer (NanoDrop ND-1000, ThermoScientific). Samples with a 260/280 purity ratio higher than ~1.8 were used for cDNA synthesis. The cDNA synthesis was performed using a qScript cDNA SuperMix kit (VWR) and the MiniOpticon Real-time PCR Detection System (MJ Mini, BioRad). All samples were normalized (1 μg of RNA in 20 μL of RNase/DNase free distilled water).

### Quantitative Real-time Polymerase Chain Reaction (qPCR)

The expression of the osteogenic genes bone morphogenic protein-2 (*BMP-2*), transcription factor *RUNX2*, and bone sialoprotein (*BSP*), and of the angiogenic genes von Willebrand factor (*vWF*), *CD31*, and vascular endothelial growth factor A (*VEGF*) were quantified in the cDNA samples using a real-time PCR reaction. The target genes were normalized with *18S rRNA* as the housekeeping. The primers (200 nM) were designed to span exon-exon junctions using the primer-BLAST tool (sequences in Table S1). The real-time PCR reaction was done using the PerfeCTa SYBR Green FastMix Reaction Mixes (Quanta Biosciences), according to manufacturer’s specifications. The reactions were monitored in a Mastercycler (Realplex4, Eppendorf) using the software realplex version 2.2 (Eppendorf). The relative quantification of the osteogenic and angiogenic markers expression was performed using the 2^−ΔΔ**CT**^ method (Perkin-Elmer). Results are expressed relatively to gene expression levels of day 3.

### Statistical Analysis

Statistical analysis was performed using two-way analysis of variance (ANOVA) with Tukey’s post-hoc test (GraphPad Prism 6.0). A p-value < 0.05 was considered statistically significant. Only non-significant differences (ns) were marked to facilitate the interpretation of the data. All results are presented as mean ± standard deviation.

## Results and Discussion

### Encapsulation of Cells within Multilayered Liquified Capsules

**Figure 2:**
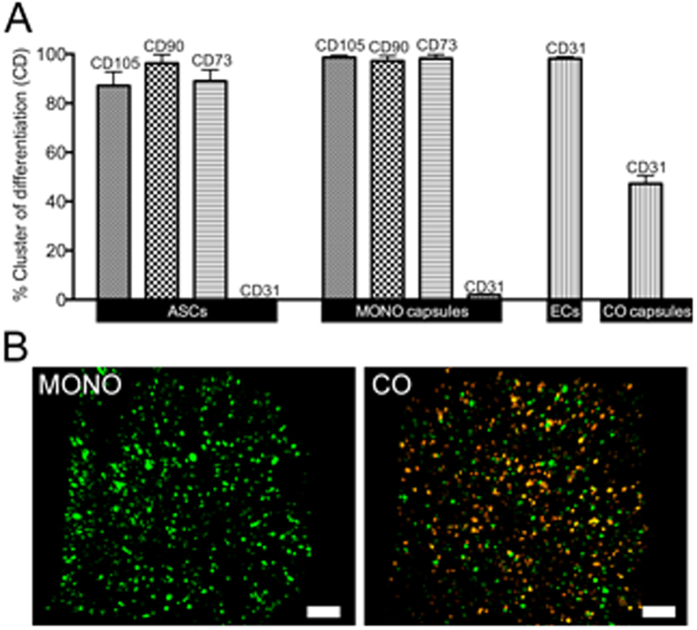
(**A**) Flow cytometry analysis of human adipose stem cells (ASCs) and human microvascular endothelial cells (ECs) after isolation and encapsulation at day 0 within MONO and CO capsules. (**B**) Confocal images of MONO and CO capsules encapsulating cells at day 0 previously labeled with DIO (green, ASCs) and DIL (orange, ECs) lipophilic fluorescent dyes (scale bar: 200 μm).

The successful isolation of ASCs and ECs from adipose tissue was determined by flow cytometry (Fig. 2A). More than 90% of the ASCs were found to be positive for the mesenchymal stem cell markers CD105, CD90, and CD73, whilst all cells lacked the expression of the endothelial marker CD31. On the other hand, the endothelial phenotype of ECs was confirmed by the CD31 expression. The maintenance of the mesenchymal and endothelial phenotypes after cell encapsulation in MONO and CO capsules also confirmed that the encapsulation process did not affect the cells. Moreover, the expression of CD31 by 47.14% ± 3.31 of the cells in the CO capsules confirmed the 1:1 ratio of the co-cultures as well as the inertness of the method. The random distribution of both cell types within the capsules was confirmed upon microscopic analysis. In the MONO capsules only green fluorescence corresponding to ASCs could be detected, while in the CO capsules both green and orange fluorescence identifying respectively ASCs and ECs could be visualized (Fig. 2B).

### Metabolic Activity, Proliferation, and Morphology of the Encapsulated Cells

**Figure 3:**
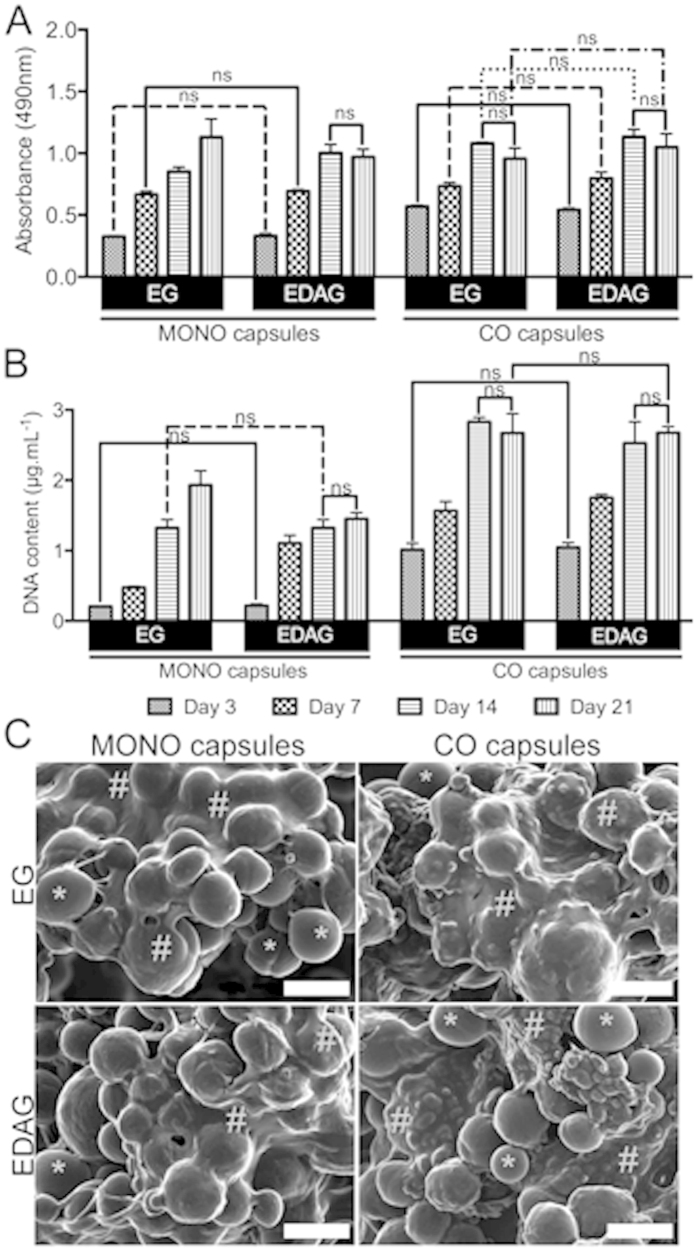
(**A**) Cell metabolic activity determined by MTS colorimetric assay and (**B**) cell proliferation by DNA quantification. All results were significantly different unless marked with ns (p > 0.05). (**C**) SEM images of the encapsulated microparticles and cells inside MONO and CO capsules after 21 days (magnification: 500×, scale bar: 50 μm). The symbols * and ^#^ mark the microparticles and the extracellular matrix deposition of the encapsulated cells, respectively.

MONO capsules in EDAG medium showed an increasing metabolic activity (Fig. 3A) and DNA content (Fig. 3B) up to 14 days of culture. However, at day 21, no significant differences were found in comparison to day 14 for both parameters. On the other hand, cells in MONO capsules cultured in EG medium kept proliferating up to the end timepoint, which was also traduced in significantly higher metabolic activity. The same trend found in MONO capsules cultured in EDAG medium was observed for the CO capsules cultured in both media. The stagnation in the metabolic activity and in the DNA content between 14 and 21 days of culture in MONO capsules cultured in EDAG medium, and in CO capsules cultured in both media might be an indication that cell differentiation is occurring. The cells interacting with the microparticles in the core of both MONO and CO capsules was visualized by SEM after 21 days of culture (Fig. 3C). In order to visualize the core contents by SEM, the layer-by-layer membrane of the capsules, which remained stable up to the 21 days of *in vitro* culture, was disrupted. In all conditions cells adhered to the surface of the microparticles, proliferated, and deposited matrix in such a way that the particles were assembled in large 3D constructs.

### *In Vitro* Assessment of the Osteogenic Potential of Liquified Multilayered Capsules

#### Quantification of ALP Activity and Mineralization Evaluation

**Figure 4:**
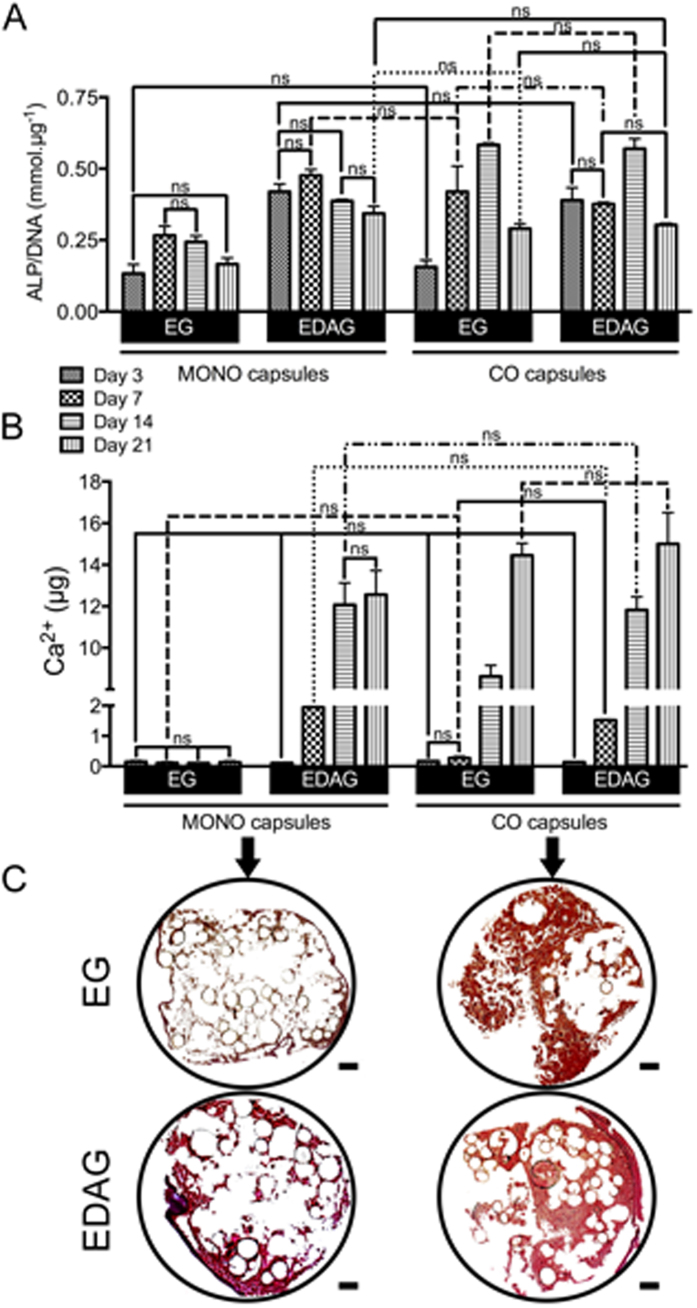
(**A**) Alkaline phosphatase (ALP) activity normalized by DNA content and (**B**) quantification of calcium. All results were significantly different unless marked with ns (p > 0.05). (**C**) Alizarin red staining on histological sections of MONO and CO capsules cultured in EG or EDAG media after 21 days. Calcium deposits were stained in red (scale bar: 50 μm).

Knowing that increased levels of ALP activity are correlated with enhanced osteogenic differentiation^31^, it can be stated that cell differentiation occurred earlier in MONO and CO capsules cultured in EDAG medium (Fig. 4A). However, as the time of culture increased, ALP activity in CO capsules cultured in EG also increased. At day 14 both CO capsules showed the highest levels of ALP activity. Therefore, co-cultures in the liquefied capsules led to an enhanced cell differentiation, although delayed when co-cultured in EG medium. MONO capsules cultured in EG medium presented the lowest ALP activity levels during the entire experiment. ALP is secreted by active osteoblasts and is responsible for the cleavage of pyrophosphate ions, which are inhibitors of the formation of hydroxyapatite crystals. The hydrolysis reaction results in the saturation of the extracellular fluid with orthophosphates that induce mineralization^32^,^33^. Hence, ALP activity results were corroborated with calcium quantification (Fig. 4B). Similarly, calcium deposits were hardly detected in MONO capsules cultured in EG medium whilst earlier detected in the capsules cultured in EDAG compared to CO capsules cultured in EG medium. In the end, co-cultures resulted in an enhanced newly deposited mineralized ECM, even in EG medium. Mineralization was qualitatively assessed by alizarin red staining at day 21 (Fig. 4C), confirming the deposition of calcium.

#### Osteopontin and CD31 Immunofluorescence Detection

**Figure 5:**
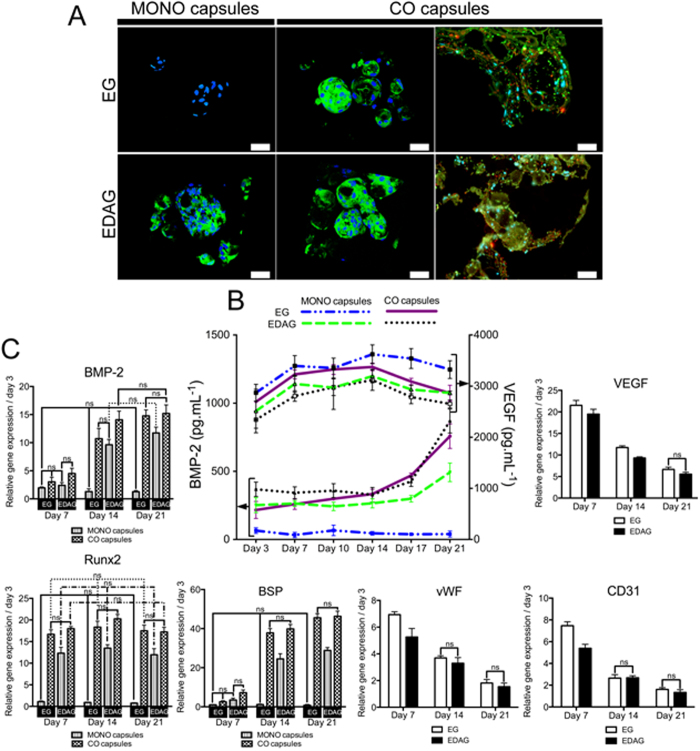
(**A**) Immunofluorescence of osteopontin (green) in MONO and CO capsules cultured in EG and EDAG media after 21 days of culture. Cells nuclei were counterstained with DAPI (blue). To visualize the encapsulated ECs, CD31 (red) was identified by immunofluorescence staining in histological sections of CO capsules cultured for 21 days (scale bar: 50 μm). (**B**) Quantification of BMP-2 and VEGF release by ELISA. (**C**) Relative expression of osteogenic (*BMP-2, RUNX2, and BSP*) and angiogenic (*VEGF, CD31*, and *vWF*) markers up to 21 days. All results were significantly different unless marked with ns (p > 0.05).

The secretion of the late osteogenic marker osteopontin in MONO and CO capsules after 21 days of culture in EG and EDAG media was confirmed (Fig. 5A). On the other hand, cells in the MONO capsules cultured in EG medium did not secrete osteopontin. The presence of the ECs in the CO capsules cultured in both media was assessed by the expression of CD31. The immunofluorescence could only be detected after histological cuts. This indicates that ECs were located in the inner regions of cell aggregates enfolded by ASCs, probably due to the higher proliferative rate of the latter. Altogether, these results indicate not only that cells were able to spread throughout the inner areas of capsules, but also that the diffusion of essential and signaling molecules for cell survival, proliferation, and differentiation was allowed. The diffusion of molecules in 3D systems is a major concern in tissue engineering strategies, since it dictates the success or failure of the regeneration process^34^. Its inefficiency can lead to tissue repair only at the circular periphery of 3D constructs, while cell death occurs in the inner areas. The liquified environment of capsules led to an excellent diffusion of molecules as already demonstrated in our previous studies^22^,^23^. The layer-by-layer methodology, that has been proposed in a variety of biomedical applications^35^, was effectively useful to produce a flexible permselective shell that sustained the viability of compartmentalized cells, while maintaining the mechanical integrity of the liquified capsules. The properties of the multilayers can be tuned to control the transfer properties of essential molecules^36^. However, the conception of a liquified environment required the introduction of solid microparticles to provide cell adhesion sites, since the encapsulated cells were anchorage dependent.

#### BMP-2 and VEGF Cytokines Release

The profile of BMP-2 and VEGF release into the supernatants of MONO and CO capsules cultured in EG or EDAG media is shown in Fig. 5B. The release of BMP-2 was similar for both MONO and CO capsules cultured in EDAG, which remained constant up to 14 days, and increase after 21 days. The highest amounts were detected for the CO capsules. On the other hand, for the CO capsules cultured in EG, BMP-2 was increasingly secreted along the entire experiment, reaching at day 14 the value detected for the CO capsules cultured in EDAG. Residual amounts of BMP-2 were detected for the MONO capsules cultured in EG during the entire experiment. The profile release of VEGF was similar for all conditions. The highest levels of VEGF were observed for MONO capsules cultured in EG, and then for CO capsules cultured in the same medium. However, as the culture time increased, the VEGF detected for CO capsules cultured in EG decreased to values similar to those observed for MONO and CO capsules cultured EDAG medium. Different studies^37^,^38^,^39^ have already been show that ASCs stimulate blood vessel growth due to the secretion of many angiogenic and anti-apoptotic growth factors, such as VEGF, which is widely known to enhance the biological performance of endothelial cells^7^,^19^,^20^. Furthermore, a positive effect on the differentiation of osteoprogenitor cells when co-cultured with endothelial cells was already documented in different studies^6^,^7^,^8^,^10^,^11^,^12^,^13^,^14^,^15^,^16^,^17^,^19^,^40^,^41^,^42^,^43^,^44^. As observed by ALP activity and calcium content results, a delayed osteogenic differentiation was observed in the CO capsules cultured in EG medium in comparison to capsules cultured in EDAG. Correlating the delay of the osteogenic differentiation in CO capsules cultured in EG medium with their initial release of higher levels of VEGF, and the absence of osteogenic differentiation in MONO capsules cultured in EG medium with their highest release of VEGF, it seems that the release of VEGF and the osteogenic stage of differentiation of the ASCs are intrinsically correlated. As time increased, while osteogenesis occurred in CO capsules cultured in EDAG the amount of VEGF decreased to similar values observed for capsules cultured in EDAG medium. These interesting findings might indicate that osteogenic differentiated ASCs have a decreased ability to enhance angiogenesis by diminishing VEGF release. A similar behavior was already reported^45^ for mesenchymal stem cells (MSCs). It was observed that MSCs cultured in a 2D cell culture system secreted the highest concentration of VEGF compared to MSCs cultured in osteogenic media, which down regulated the gene expression of pro-angiogenic factors. Therefore, although the ultimate goal of the proposed bioencapsulation system is to promote the osteogenic differentiation, the delayed osteogenesis observed in CO capsules in EG medium could represent an advantage - the higher amounts of VEGF released by those capsules in the initial times of culture can lead to an enhanced angiogenesis and, consequently, to a vascularized osteogenic tissue. Importantly, besides inducing angiogenesis, VEGF is also known to increase the osteogenic healing capacity of ASCs^46^. Likewise, Boletreau *et al.*^47^ showed that microvascular endothelial cells were able to release BMP-2 by the action of VEGF treatment. Although MONO capsules cultured in EDAG medium also released BMP-2 during the entire experiment, this was enhanced by co-encapsulation. Interestingly, when the release of VEGF started to decrease in both CO capsules, the release of BMP-2 increased exponentially. In this case, the synergistic effect of the co-cultures and the presence of osteogenic differentiation factors in the culture medium resulted in the highest BMP-2 release by CO capsules cultured in EDAG medium. However, in later timepoints the co-culture was sufficient to stimulate BMP-2 release since the amount released by CO capsules cultured in EG was similar to the one observed for the EDAG. Since VEGF and BMP-2 were detected and measured in the culture medium surrounding the capsules, this indicates that the membrane is permeable to small molecules while remaining stable up to 21 days of *in vitro* culture. We envisage that after implantation, the developed capsules can positively influence the neighboring cells by paracrine signaling in the highly vascularized environment of bone.

#### Genetic Profile Quantification of Osteogenic and Angiogenic Markers

The quantification of the transcripts for the osteogenic and angiogenic genes of interest was performed at the different timepoints (Fig. 5C). The expression of the osteogenic genes *BMP-2, BSP, and RUNX2*, was up regulated in all capsules and at all the timepoints, excepting for MONO capsules cultured in EG media. While for *BMP-2* and *BSP* markers, the expression level increased with culture time, for *RUNX2* no significant differences were found over time, although a slight peak could be observed at day 14. Again, no significant and distinct results were found between CO capsules cultured in EG and EDAG media, indicating that the crosstalk between the two cell types was sufficient to induce the osteogenic differentiation. In all CO capsules the endothelial markers *vWF*, *CD31*, and *VEGF* were up regulated up to 21 days although its expression decreased with culture time, as similarly observed in co-culture systems^8^. This decrease might be explained by the fact that total RNA was used to normalize the genetic profile results, thus including RNA also from ASCs and not only from ECs. Significant differences were found for the expression of *VEGF* marker at days 7 and 14, and for *vWF* and *CD31* markers at day 7, at which CO capsules cultured in EG medium presented the highest expression.

Altogether these results showed that the crosstalk between ASCs and ECs played a significant role in osteogenic differentiation. More importantly, the co-encapsulation of ASCs and ECs suppressed the need of dexamethasone and ascorbic acid to osteoblastic differentiation occurs. Considering the reported findings, we believe that the proposed CO capsules will find great applicability in bone regeneration, while bringing new insights to bioencapsulation strategies. Additionally, in the present study a new 3D biomimetic platform to assess the interaction of stem and endothelial cells was developed. Importantly, the biological outcome demonstrated was achieved by using cells isolated from a waste and abundant tissue, which is highly desirable for tissue engineering applications considering the high number of cells required in 3D systems. Since it was demonstrated that the capsules also release important cytokines for bone healing, namely VEGF and BMP-2, we thus believe to be close to a completely self-regulated confined system that could stimulate autonomous production mineralized bone tissue, with clear therapeutic advantages. The liquified environment of capsules is a key point in the novelty and success of the proposed system, since it allows the encapsulated cells to freely move and proliferate – as they can easily occupy the liquid space of the capsules environment - and self-organize a 3D culture composed by microparticles. Consequently, we envisage that in a dynamic culture the results obtained in the present study could be boosted by enhancing the diffusion of molecules across the liquified environment, such as essential molecules for cells survival or even signaling biomolecules either circulating outside the capsules environment or released by the encapsulated cells. Additionally, we trust that minimal invasive procedures could be adopted due to their injectability provided by the liquified environment. Moreover, having a single structure encapsulating all the different components might facilitate the implantation process, while avoiding their dispersion to other regions of the body. This study focused its applicability in the bone regeneration field, but we believe that such kind of complex hierarchical systems able to encapsulate multi-phenotypic cells could find important applications in other TE strategies, such as closed devices to be used in drug screening or disease models.

## Conclusion

Inspired by the local interactions at the molecular level from the co-existence of stem and vascular cells in the native environment of bone, we developed a stem-cell based bioencapsulation strategy. The co-encapsulation of stem and endothelial cells, both derived from adipose tissue, within a single hierarchical structure proved to be an effective strategy for the *in vitro* osteogenic differentiation. Overall, the co-encapsulation of ECs lead ASCs to differentiate into the osteogenic lineage inside the liquified and multilayered capsules with microparticles, even in the absence of two major osteogenic differentiation factors, namely dexamethasone and ascorbic acid. Additionally, CO capsules released BMP-2 and VEGF, which evidences the potential of the capsules as a cytokine delivery system. We believe that the developed co-encapsulation strategy represents a ground-breaking advance in the engineering of 3D compartmentalized devices that aim to control the cascade of biological processes and lead to faster high-quality new tissue formation with minimum cell manipulation.

## Additional Information

**How to cite this article**: Correia, C. R. *et al.* Semipermeable Capsules Wrapping a Multifunctional and Self-regulated Co-culture Microenvironment for Osteogenic Differentiation. *Sci. Rep.* **6**, 21883; doi: 10.1038/srep21883 (2016).

## Supplementary Material

Supplementary Information

## Figures and Tables

**Figure 1:**
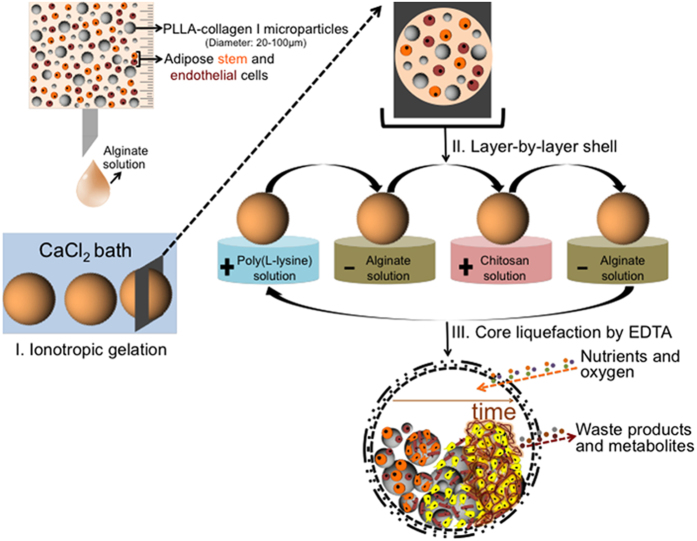
Production of the proposed liquified multilayered capsules encapsulating poly(L-lactic acid) microparticles coated with collagen I, and adipose stem (orange, ASCs) and endothelial cells (red). The loaded hydrogel particles are obtained after the ionotropic gelation of alginate in a calcium chloride (CaCl_2_) bath. Then, layer-by-layer is performed with the polyelectrolytes, namely poly(L-lysine), alginate, and chitosan, in order to produce the multilayered membrane. Ultimately, the liquified core is obtained by chelation with EDTA. As the time of culture increases, the encapsulated cells subsequently adhere to the surface of microparticles, proliferate, and create cell aggregates. The ASCs start to differentiate into the osteogenic lineage (color change from orange to yellow) and ultimately a mineralized osteogenic matrix is obtained inside the liquified environment of capsules. The multilayered membrane allows the exchange of essential molecules for cell survival.
