# Supplementary material for: Semipermeable Capsules Wrapping a Multifunctional and Self-regulated Co-culture Microenvironment for Osteogenic Differentiation
Source: Sci Rep. 2016 Feb 24;6:21883. doi: 10.1038/srep21883 (PMC4764811; doi:10.1038/srep21883)
Supplement: Supplementary Information [file srep21883-s1.pdf]

Supplementary information

Semipermeable Capsules Wrapping a Multifunctional and Self-regulated Co-culture Microenvironment for Osteogenic Differentiation

Clara R. Correia[1,2], Rogério P. Pirraco[1,2], Mariana T. Cerqueira[1,2], Alexandra P. Marques[1,2], Rui L. Reis[1,2], João F. Mano[1,2]*

[1]3B's Research Group – Biomaterials, Biodegradables and Biomimetics, University of Minho, Headquarters of the European Institute of Excellence on Tissue Engineering and Regenerative Medicine, AvePark, 4805-017 Barco, Guimarães, Portugal
[2]ICVS/3B's – PT Government Associate Laboratory, Braga/Guimarães, Portugal
[*]Corresponding author. Fax: 00351253510909. Email: jmano@dep.uminho.pt

Table S1 – Primer sequences used for real-time polymerase chain reaction.

**Housekeeping**	18S	Forward (5'-3')	GAAACCTTCCGACCCCTCTC
		Reverse (5'-3')	TACGAGGTCGATTTGGCGAG
**Osteogenic markers**	BMP-2	Forward (5'-3')	TGAATCAGAATGCAAGCAGG
		Reverse (5'-3')	TCTTTTGTGGAGAGGATGCC
	RUNX2	Forward (5'-3')	TTCCAGACCAGCAGCACTC
		Reverse (5'-3')	CAGCGTCAACACCATCATTC
	BSP	Forward (5'-3')	ACTGAGCCTGTGTCTTGAAA
		Reverse (5'-3')	CTTCCAACAGCCAATCACTG
**Angiogenic markers**	vWF	Forward (5'-3')	CCCTGGGTACAAGGAAGAAAAT
		Reverse (5'-3')	AGTGTCATGATCTGTCTCCTCTTAG
	CD31	Forward (5'-3')	AAGGCCAGATGCACATCC
		Reverse (5'-3')	TTCTACCCAACATTAACTTAGCAGG
	VEGF	Forward (5'-3')	GATCCGCAGACGTGTAAATG
		Reverse (5'-3')	TACGAGGTCGATTTGGCGAG
